# Dispersal and group formation dynamics in a rare and endangered temperate forest bat (*Nyctalus lasiopterus,* Chiroptera: Vespertilionidae)

**DOI:** 10.1002/ece3.2330

**Published:** 2016-10-20

**Authors:** João D. Santos, Christoph F. J. Meyer, Carlos Ibáñez, Ana G. Popa‐Lisseanu, Javier Juste

**Affiliations:** ^1^ Centre de Coopération Internationale en Recherche Agronomique pour le DéveloppementAvenue Agropolis34398MontpellierFrance; ^2^ Centre for Ecology, Evolution and Environmental Changes (cE3c)Faculty of SciencesUniversity of Lisbon1749‐016LisbonPortugal; ^3^ School of Environment and Life SciencesUniversity of SalfordSalfordM5 4WTUnited Kingdom; ^4^ Department of Evolutionary EcologyEstación Biológica de Doñana (CSIC)Avenida Américo Vespucio s/n41092SevilleSpain

**Keywords:** Colony formation, genetic structure, microsatellites, mtDNA, philopatry, relatedness

## Abstract

For elusive mammals like bats, colonization of new areas and colony formation are poorly understood, as is their relationship with the genetic structure of populations. Understanding dispersal and group formation behaviors is critical not only for a better comprehension of mammalian social dynamics, but also for guiding conservation efforts of rare and endangered species. Using nuclear and mitochondrial markers, we studied patterns of genetic diversity and differentiation among and within breeding colonies of giant noctule bats (*Nyctalus lasiopterus*), their relation to a new colony still in formation, and the impact of this ongoing process on the regionwide genetic makeup. Nuclear differentiation among colonies was relatively low and mostly nonsignificant. Mitochondrial variation followed this pattern, contrasting with findings for other temperate bat species. Our results suggest that this may indicate a recent population expansion. On average, female giant noctules were not more closely related to other colony members than to foreign individuals. This was also true for members of the newly forming colony and those of another, older group sampled shortly after its formation, suggesting that contrary to findings for other temperate bats, giant noctule colonies are not founded by relatives. However, mother–daughter pairs were found in the same populations more often than expected under random dispersal. Given this indication of philopatry, the lack of mitochondrial differentiation among most colonies in the region is probably due to the combination of a recent population expansion and group formation events.

## Introduction

Studying natural populations in their habitat can prove difficult using traditional methods such as mark‐recapture and radiotelemetry (Clutton‐Brock and Lukas 2012). This is particularly true when studying the dispersal habits of small, highly mobile and nocturnal animals such as bats. Furthermore, these methods provide estimates of individual mobility and dispersal, but not of their effective rate at the population level (Prugnolle and de Meeûs 2002). In contrast, genetic methods that allow inferring the distribution of alleles across populations can provide estimates of gene flow, and thus information on the reproductive success of migrating individuals (Wright 1943; Slatkin 1987). The genetic structure of natural populations can result from a number of interacting factors, such as recent history, dispersal, mating system and group formation (Chesser 1991; Storz 1999; Parreira and Chikhi 2015). Dispersal ability in particular has been shown to be negatively correlated with genetic differentiation across a range of taxa (e.g., plants, Govindaraju 1988; mammals, Bohonak 1999), including temperate bats, where genetic population structure correlates negatively with the extent of migration (Moussy et al. 2013; Burns and Broders 2014).

The formation of a new colony or social group is a rarely witnessed process that is particularly interesting for its effect on regionwide genetic variation and for providing information about the underlying social dynamics. Where groups consist of philopatric adults, the formation of a new group is usually the result of group fission (Alberts and Altmann 1995; Hoogland 1995; Thierry 2007; Kerth 2008; Armitage et al. 2011). However, the level of kinship among the members of the resulting groups varies across species. While in Savannah baboons (*Papio cynocephalus*) social bonds can supersede kin relations in the choice between emerging groups (Van Horn et al. 2007), for a range of other primate species (Van Horn et al. 2007; Snyder‐Mackler et al. 2014), as well as African elephants (*Loxodonta africana,* Archie et al. 2006), hyenas (*Crocutta crocutta*, Holekamp et al. 1993) and yellow‐bellied marmots (*Marmota flaviventris*, Armitage 1987), females choose to remain or move together with close kin. The latter has also been documented for big brown bats, *Eptesicus fuscus*, in which average pairwise relatedness was higher than expected among individuals of three of five matrilines following the formation of a new group (Metheny et al. 2008). Previous studies had found little or no correlation between the degree of association and relatedness levels among members of bat maternity colonies, including in this particular species (Kerth and König 1999; Metheny et al. 2007). These estimates had, however, been obtained from established colonies. During colonization, higher levels of relatedness would likely facilitate cooperative behaviors, counterbalancing the increased risk incurred (Greenwood 1980). Nevertheless, the structure and relationships within any group will be shaped by the composition of its founders, socially as well as genetically.

**Figure 1:**
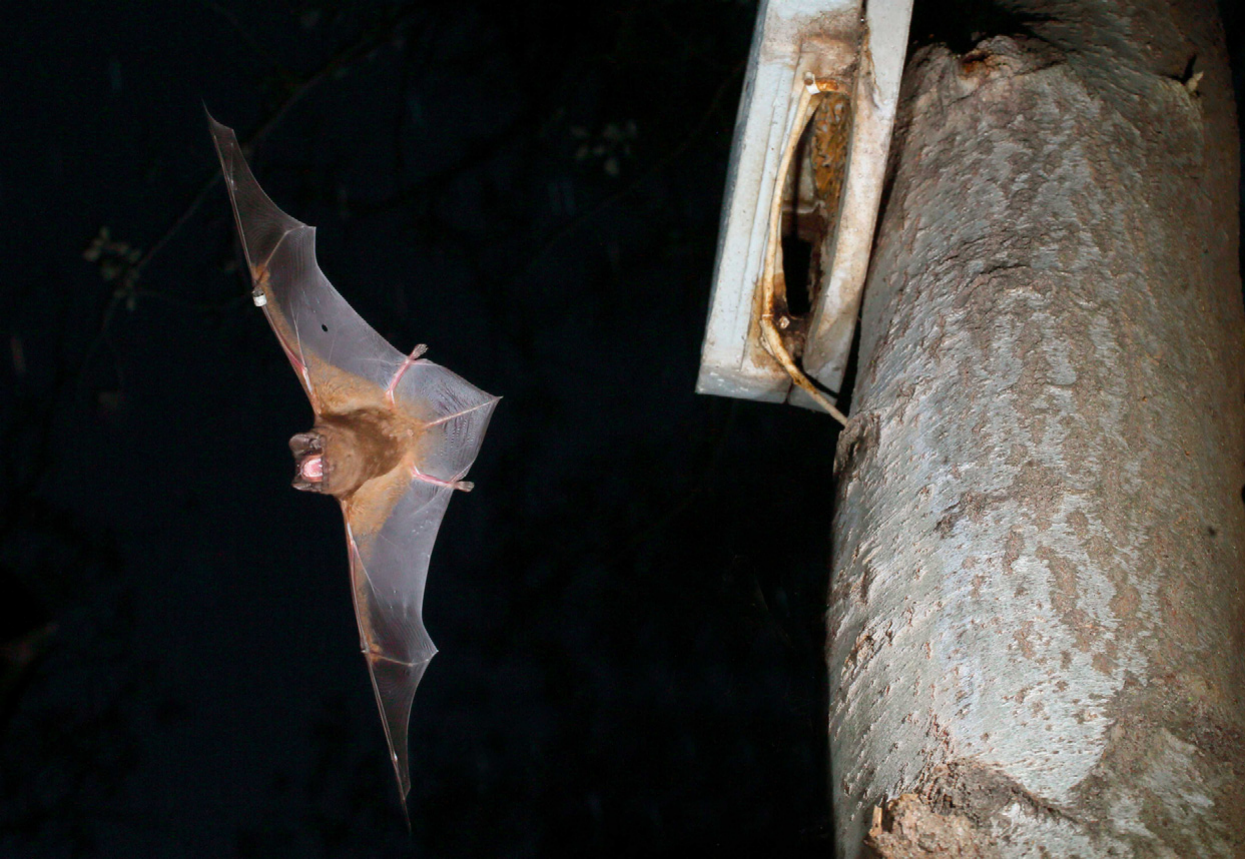
Giant noctule bat, *Nyctalus lasiopterus*, as it leaves the roost at dusk in the newly forming colony in Doñana National Park. Photo: Jens Rydell.

The giant noctule, *Nyctalus lasiopterus*, with a wingspan of up to 45 cm and weighing around 50 g, is the largest European bat species (Ibáñez et al. 2004; Fig. 1). It is also one of the rarest, with only a few known breeding colonies in Spain, Hungary, and France (Ibáñez et al. 2004; Estók 2007; Hutson et al. 2008; Dubourg‐Savage et al. 2013). A tree‐roosting species, the giant noctule has a patchy circum‐Mediterranean distribution throughout southern Europe (Iberia, France, Italy, the Balkans and Greece), North Africa, and Anatolia. The species’ range also extends into the Caucasus, Iran, Kazakhstan, and the Urals (Ibáñez et al. 2004). The demographic dynamics observed in the Iberian Peninsula (Ibáñez et al. 2009) indicate that, similar to other temperate bats, giant noctule bats segregate sexually during spring and summer to form breeding colonies (Bradbury 1977; McCracken and Wilkinson 2000). These aggregations of giant noctule females form fission–fusion societies akin to those described for other temperate forest bats (Kerth and König 1999; Willis and Brigham 2004; Patriquin et al. 2013) in which frequent roost changes result in nonrandom associations between colony members (Popa‐Lisseanu et al. 2008). The benefits of this social system and the factors underlying the individual decisions behind it are still under debate (Aureli et al. 2008; Sueur et al. 2011).

Colonization of new areas and the formation of new colonies are rare events that have seldom been described in bats, and on only one occasion has colonization been studied in detail from a genetic perspective (*Eptesicus fuscus,* Metheny et al. 2008). As part of a long‐term study of giant noctule populations in southwestern Andalusia, Spain, we examined the influence of genetic relatedness on the formation of a new colony in Doñana National Park prior to 2007 and after 2010, following a temporary, unexplained 3‐year abandonment. We sampled individuals regularly roosting in this new colony, in addition to three stable breeding colonies in the region. Using both nuclear and mitochondrial markers, we assessed genetic population structure and levels of genetic relatedness within colonies. To test the hypothesis that the colonizer group was kin‐based, that is, that the foundation of this new group was the result of a joint movement of related females, we first determined whether among‐group genetic variance had increased after the establishment of this new colony. Subsequently, we estimated genetically inferred relatedness and putative relations among individuals within colonies and within matrilines. We predicted higher levels of relatedness among colonizing females in Doñana National Park than expected by chance. Likewise, if related females moved together, we expected to find higher levels of average pairwise relatedness among females of the same matriline in the new group when compared to females carrying the same haplotypes in other colonies.

We discuss the implications of our findings with regard to the social habits of giant noctules and their demographic history in the region and, in a more general context, as to how they advance our understanding of mammalian social structure and the role played by kinship in the formation of new colonies.

## Materials and Methods

### Study populations and sampling

**Figure 2:**
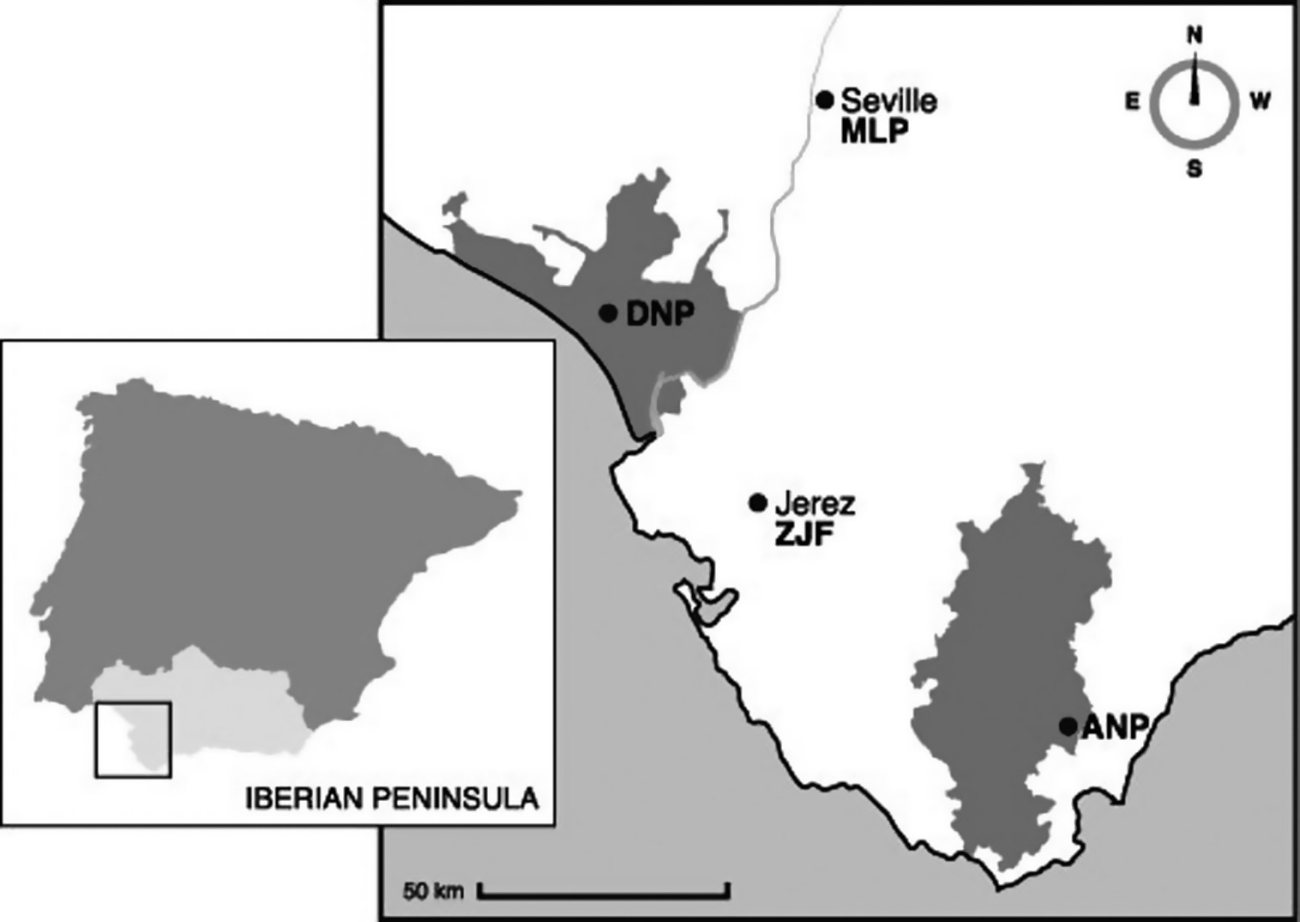
Location of the three maternity colonies and colonization site included in the study, as well as major towns and rivers. Grey areas indicate Natural and National parks of “Los Alcornocales” and “Doñana”, respectively.

We sampled a total of 215 individuals present in four maternity colonies in southern Andalusia, Spain. The breeding colony in Doñana National Park (DNP) is located around a group of bat boxes in a small stand of mainly Eucalyptus trees near the marshes at the mouth of the Guadalquivir River (36.99° N, 6.44° W). Two breeding colonies of *N. lasiopterus* were recently reported from southwestern Andalusia (Ibáñez et al. 2009; Fig. 2): one in large, old plane trees (*Platanus* sp.) within “Maria Luisa Park” (MLP) in the city of Seville (37.37° N, 5.59° W). This was the larger of the two colonies, with an estimated 500 bats roosting there in 2007 (Popa‐Lisseanu et al. 2008). The other colony occupied a group of palm trees (*Washingtonia* sp.) located in the gardens of the zoo of Jerez de la Frontera (ZJF; 36.70° N, 6.15° W); this colony had an estimated population of 100–150 females. In contrast to these seminatural colonies, the fourth population is found in a large natural Mediterranean mixed oak forest in “Los Alcornocales Natural Park” (ANP) around 100–150 km southeast of Seville (36.31° N, 5.44° W) and has an estimated size of several thousand individuals that were sampled at different localities.

Samples consisted of wing punch biopsies (Worthington Wilmer and Barratt 1996) stored in 70% ethanol. We analyzed 84 samples from MLP, 52 from ANP and 32 individuals from ZJF. A total of 47 individuals were sampled from the newly forming colony in DNP. This data set was split into: (1) the Doñana “original” colonizing group (D_O_; *N* = 23), consisting of samples collected between 2003 and 2005; and (2) the Doñana “recolonization” group (D_R_; *N* = 24), sampled after the yet unexplained three‐year breakdown (2007–2009), during the subsequent recolonization process from 2010 to 2013. For both D_O_ and D_R_, we selected only females that were registered breeding in the colony during more than 1 year.

### Molecular markers

Total genomic DNA was extracted from wing punches using a modified salt‐based protocol (Aljanabi and Martinez 1997). The two hypervariable domains (HVI and HVII) of the mitochondrial control region were PCR‐amplified using primers L15926 (Kocher et al. 1989) and CSBF‐R (Wilkinson and Chapman 1991) for HVI, and L16517 (Fumagalli et al. 1996) and H607 (Worthington Wilmer et al. 1994) for HVII (forward and reverse primers, respectively). Sequences were aligned, visually inspected for ambiguities, and edited by hand using Sequencher v 4.9 (Gene Codes Corp., Ann Arbor, MI). The final sequences were cropped to a length of 437 bp for HVI (including the initial sequence and first repeat of the HVI region as well as flanking tRNA genes and part of the cyt b gene) and 397 bp for HVII.

All individuals were additionally genotyped at 11 nuclear microsatellite loci. As no specific microsatellites yet existed for *N. lasiopterus*, annealing temperatures and PCR mix concentrations were optimized for eight markers developed for *N. leisleri* (Nle 2,3, and 6–11; Boston et al. 2008), one developed for *Eptesicus fuscus* (EF4, Vonhof et al. 2002) and two developed for *Nyctalus noctula* (P20, P217; Mayer 1997). All were tested in muscle tissue prior to genotyping. Labelling followed Schuelke's procedure (2000).

See Appendix S1 for a detailed description of DNA extraction, amplification, sequencing, and microsatellite genotyping.

### Data analysis

#### Mitochondrial DNA

The two mitochondrial fragments were concatenated and the number of haplotypes (*h*), haplotype diversity (*Hd*), nucleotide diversity (*π*), and the number of segregating sites (*S*) were calculated using DNASP v. 5.10.1 (Rozas 2009). A median‐joining network based on haplotypes was constructed using NETWORK (Bandelt et al. 1999). Through analyses of molecular variance (AMOVA, Excoffier et al. 1992), we assessed how genetic variation was partitioned among colonies, whereby we explored different grouping combinations to identify the one that maximized the among‐group component of genetic variation. AMOVA was performed using the software ARLEQUIN v. 3.5.1.2 (Excoffier et al. 2005), which was also used to calculate *ɸ*_ST_ values among colonies.

#### Microsatellites

All microsatellite loci were tested for genotyping errors using MICROCHECKER v. 2.2.3 (Van Oosterhout et al. 2004). Linkage disequilibrium among markers was assessed using FSTAT v. 2.8.3.2 (Goudet 2001). Identification of loci under selection was performed using the software ARLEQUIN v. 3.5.1.2. Calculations of allele frequencies (including null alleles) across colonies, observed (*H*_*o*_) and expected (*H*_*e*_) heterozygosities, as well as deviations from Hardy–Weinberg equilibrium (HWE) were performed in CERVUS v. 3.0.6 (Kalinowski et al. 2007). Allelic richness was assessed using the R package “hierfstat” (Goudet 2005).

Given the recent developments and ongoing debate about the various existing population differentiation measures and their appropriate use (Hedrick 1999; Jost 2008; Heller and Siegismund 2009; Meirmans and Hedrick 2011), we opted to estimate both D_EST_ and *F*_ST_, the former for a more robust analysis and as a reference for future studies, the latter to facilitate comparison with results from previous studies. Both measures were calculated using the R package “diveRsity” (Keenan et al. 2013). As for mtDNA, partitioning of genetic variation at the nuclear level was assessed with AMOVA in ARLEQUIN 3.5.1.2.

#### Genetic relatedness

Pairwise and mean relatedness values (*R*), both among colonies and for matrilines (between individuals with shared mitochondrial haplotypes), were estimated using ML‐Relate (Kalinowski et al. 2006). This software implements a corrected maximum‐likelihood approach that allows loci with null alleles to be incorporated into the analysis (Wagner et al. 2006). Mother–daughter pairs were identified, allowing not only to determine the number and proportion of close kin (*r *>* *0.25) and of mother–daughter pairs within our data set, but also to examine the distribution of these dyads across colonies. Assignments inconsistent with mitochondrial haplotypes were excluded. For each colony, we estimated the proportion of close associations out of all possible pairs of individuals (% *r* > 0.25), as well as the proportion (%) of females with at least one close relative within the colony.

## Results

### Genetic diversity

**Figure 3:**
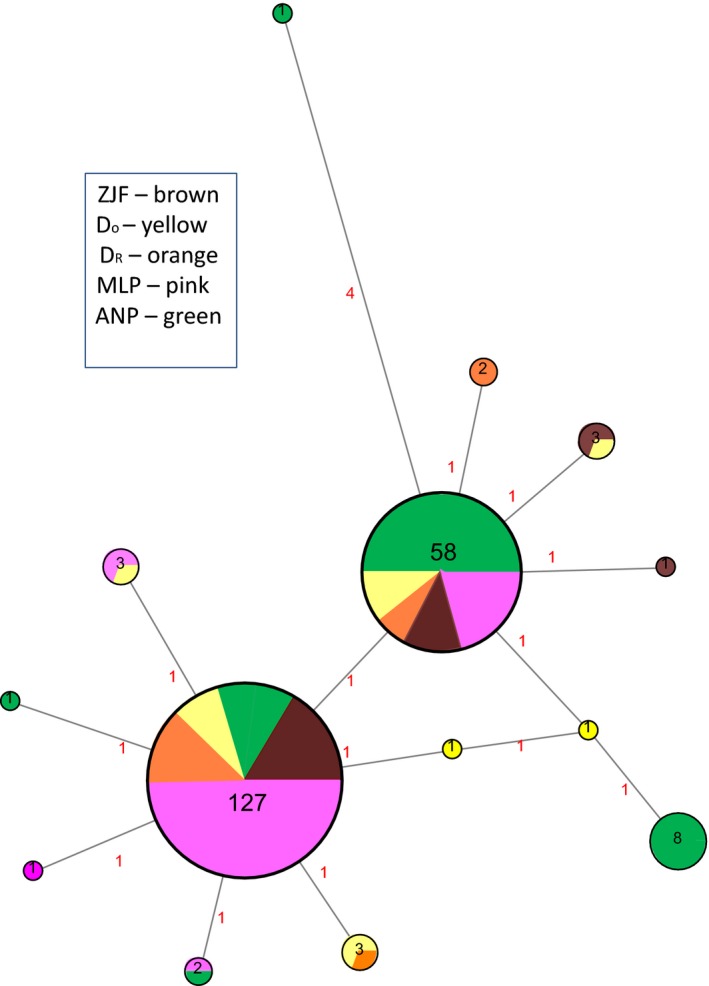
Parsimony‐based network of mtDNA haplotypes using the median‐joining algorithm. Circles correspond to haplotypes with size proportional to the number of individuals sharing this particular haplotype. Colors correspond to the four colonies/populations studied (see text for acronyms), and red numbers indicate the number of mutational steps needed to connect the haplotypes.

A total of 15 haplotypes were found, which varied on average by only one substitution, comprising a total of 15 polymorphic sites. The two most common haplotypes were present in all colonies (Fig. 3) and together represented 86% of the individuals sampled. The remaining 13 haplotypes were found in two populations at most, six of them being present in only one. Colonies had between 4 and 8 haplotypes (mean 5.6 ±  SD 1.52). Haplotype diversity ranged from 0.179 to 0.759 (total Hd = 0.578, Table 1), being lowest for ZJF and highest for D_O_ (first colonization attempt of Doñana), followed by ANP (the only two colonies located in a “natural” habitat). The median‐joining network showed a star‐shaped structure around the two most frequent haplotypes (Fig. 3).

**Table 1:** Genetic diversity in the mitochondrial and nuclear markers across all loci and by colony. The number of individuals sampled (*N*) and the variation in sampling time (*S*_var_) are also given. [number of haplotypes (*h*), haplotype diversity (*Hd*), nucleotide diversity (*π*), number of polymorphic sites (*S*), observed (*Ho*) and expected (*He*) heterozygosity]

			Mitochondrial				Nuclear	
Colony	*N*	*S*_var_	*h*	*Hd*	*π*	*S*	*He*	*Ho*
D_O_	23	–	8	0.759	0.00128	6	0.666	0.625
D_R_	24	–	4	0.498	0.00079	3	0.681	0.680
MLP	84	1.24	6	0.354	0.00012	3	0.747	0.684
ANP	52	1.3	5	0.614	0.00135	8	0.761	0.647
ZJF	32	0.47	5	0.179	0.00022	2	0.787	0.675
Total	215	2.10	15	0.578	0.00042	14	0.761	0.647

All microsatellite loci were polymorphic, with an average of 12 alleles, and all were in linkage equilibrium. *H*_*o*_ ranged from 0.347 to 0.850 (Table S1). Out of the 11 microsatellites, four (Nle9, Nle11, P20 and P217, see Table S1) deviated significantly from HWE and were excluded from the genetic structure analysis. Selection acting on Nle11 could not be ruled out (*P* < 0.05), further supporting its exclusion. For estimating relatedness, we resorted to Wagner et al.'s (2006) method, implemented in ML‐Relate, and kept all 11 loci.

### Population differentiation

Mitochondrial differentiation according to *ɸ*_ST_ averaged 0.11 ± 0.12 (range 0–0.36). This value was due mainly to ANP, which differed significantly from all other colonies (Table 2). For microsatellites, pairwise *F*_ST_ values between DNP's first colonization attempt (D_O_) and the three other colonies were on average low (0.015 ± 0.01), ranging between 0 and 0.035 (Table 2). Significant pairwise differences among colonies, nonetheless, separated ANP from D_O_, MLP, and D_R_. Estimated values of population differentiation using D_EST_ (Table S2) differed slightly from those based on *F*_ST_, yet both measures were significantly correlated (*R* ^2^ = 0.46, *P* = 0.03). Nevertheless, no pairwise comparisons based on D_EST_ were significant.

**Table 2:** Pairwise *F*_ST_ (above diagonal, microsatellite data) and *ϕ*_ST_ (below diagonal, mtDNA) values among colonies of giant noctule bats in Andalusia, including Doñana's “original” (D_O_) and “recolonization” (D_R_) groups

Colony	D_O_	D_R_	MLP	ANP	ZJF
D_O_	–	0	0.0177	**0.0351**	0.0188
D_R_	0.030	–	0.0111	**0.0267**	0.0098
MLP	**0.143**	0.003	–	**0.0093**	0.0017
ANP	**0.085**	**0.228**	**0.356**	–	0.0027
ZJF	0.029	0	0.011	**0.213**	–

Significant values (*P* < 0.05) are in bold; see text for population acronyms.

The largest proportion of mitochondrial genetic variation was explained by the within‐group component (mean = 76.83%, SD = 3.02), whether DNP was included or not. Among‐colony variation (among‐group component) showed a slight decrease when either D_O_ or D_R_ were included in the analysis (Table 3). Exploring different grouping designs, we found that among‐group variation was maximized when ANP was kept isolated, and MLP and ZJF united (I and III; Table 4). Again, this proved true, whether DNP was included or not. Adding either of the colonizer groups resulted in lower among‐colony variation, whereas this component was maximized when the two colonizer groups were grouped together with MLP and ZJF (II, III; Table 4). Nuclear variation was not affected by the different grouping strategies, with values of the among‐group component always below 1% (Table 4).

**Table 3:** Partitioning of mitochondrial genetic variation among and within colonies of giant noctule bats in Andalusia, Spain. Genetic variation components were calculated without DNP, with D_O_ without D_R_, and with D_R_ without D_O_. All other colonies (ZJF, ANP, MLP) were kept separate. Contributions of “among” and “within” components given as percentage of the total variation

Source of variation	DNP excluded	Following 1st colonization attempt (D_O_)	Following 2nd colonization attempt (D_R_)
Among colonies	26.5	20.6	22.4
Within colonies	73.5	76.4	77.6
*P*‐value	<0.001	<0.001	<0.001

**Table 4:** AMOVA‐estimated variance components among colonies of giant noctule bats in Andalusia, Spain, according to different grouping designs. Contributions of the different variance components are given as percentage of total variation

	Group I		Group II		Group III		Group IV	
	mtDNA	nDNA	mtDNA	nDNA	mtDNA	nDNA	mtDNA	nDNA
Among groups	31.2	0.62	27.5	0.71	31.5	0.58	3.05	0.0
Among populations within groups	2.36	0.33	2.39	0.47	1.27	0.71	15.89	1.32
Within populations	66.45	99.05	70.1	98.82	67.27	98.72	81.0599.14	99.14
*F*_CT_	0.311	0.006	0.274	0.007	0.314	0.006	0.031	0.000
*F*_ST_	0.335***	0.009***	0.299***	0.012***	0.327***	0.012***	0.189***	0.009***
*F*_SC_	0.034	0.003	0.033*	0.004	0.018	0.007***	0.163***	0.013**

Grouping structure: Group I: [MLP‐ZFJ]‐[ANP]; Group II: [MLP‐ZFJ‐Do]‐[ANP]; Group III: [MLP‐ZFJ‐D_R_]‐[ANP]; Group IV: [ANP‐ZFJ‐Do‐D_R_]‐[MLP].

Significant fixation indices are also shown (*P < 0.05, **P < 0.01, ***P < 0.001).

### Relatedness estimates

Mean pairwise relatedness within colonies was very low (0.075 ± 0.10, Table 5). Average relatedness values within matrilines in the different colonies varied considerably but were altogether also low (0.055 ± 7e‐2, Table 6), ranging from 0 (D_R_, H2) to 0.345 (MLP, H5), although the latter consisted of only two females. Of the four haplotypes found in D_R_, one was carried by only one female and two by unrelated females (H2 and H3, Table 6). Finally, average pairwise relatedness among females sharing H1 was low, with only three of its females being closely related (*r* > 0.25, Table 6). The number of females with at least one close relative in the same colony was high (62.5–93%, Table 5). Here, ZJF and ANP presented the lowest averages, 62.5 and 80.8%, respectively. Relationship estimates based on microsatellite data revealed an elevated number of parental associations across all populations that involved approximately half the individuals sampled (57.1%, *N* = 215, Table 5). As many as 72.7% of all paired females originated from the same colony. In MLP, this resulted in 43 of the 84 individuals (51%) roosting with their putative mothers/daughters. In ANP, 13 parental associations (involving 21 females, 40%) were found, while in DNP we only identified four (all within the post‐2007 group). No such association was found among individuals from ZJF. As for inferred mother–daughter dyads pairing females from DNP together with females from other colonies, we found five involving females from D_O_, and 12 involving females from D_R_. Regarding the former, three of five involved females from ANP (the two others assigned to MLP and ZJF), while in the latter, 9 of 12 dyads involved females from MLP (two involved the same female from ZJF, the last one ANP). The number of mother–daughter pairs was uncorrelated with variation in sampling year for each colony (*R* ^2^ = 0.0, *P* = 053), but increased significantly with the number of samples of each colony (*R* ^2^ = 0.90, *P* = 0.009).

**Table 5:** Mean pairwise relatedness *R* within colonies, percentage of closely related dyads, percentage of females with close relatives within colonies, and number of parental associations per population (*n*_par_)

Colony	*R* (mean ± SD)	% associations with *r *>* *0.25	% females with close relatives	*n*_par_
D_O_	0.046 (±0.090)	1.3	83.3	0
D_R_	0.040 (±0.078)	1.0	91.3	4
DNP	0.085 (±0.109)	9.5	93.6.5	4
MLP	0.059 (±0.097)	6.6	97.6	39
ANP	0.052 (±0.091)	6.0	80.8	11
ZJF	0.048 (±0.076)	3.4	62.5	0
Total	0.059 (±0.090)	6.1	1	105

**Table 6:** Average pairwise relatedness (±SD) among individuals with shared mitochondrial haplotypes roosting in the same colony, as well as the percentage of individuals found in any particular colony (columns) carrying a specific haplotype (rows). Only haplotypes carried by at least two individuals in the same colony are given. See text for the acronyms of the localities

Haplotypes	MLP	ANP	ZJF	D_O_	D_R_
H1	0.062 (0.102) 52%	0.027 (0.0556) 10.4%	0.046 (0.071) 16.8%	0.030 (0.068) 8%	0.039 (0.073) 4.8%
H2	0.064 (0.141) 20.7%	0.052 (0.0955) 50%	0.009 (0.033) 12.1%	0.0183 (0.035) 10.3%	0.00 6.9%
H3	–	–	–	–	0.00 100%
H4	–	–	0 66.6%	–	–
H5	0.345 66.6%	–	–	–	–
H6	–	0.023 (0.110) 100%	–	–	–

## Discussion

### Population structure and recent demographic expansion

We genotyped bats from three consolidated colonies and a recently colonized site (with two colonization events) and assayed variation both at nuclear and mitochondrial loci and levels of differentiation among the colonies. Haplotype diversity was highest in the D_O_ and ANP colonies, both situated in natural environments, whereas the two other stable colonies are located in urban parks. Mitochondrial differentiation and, to a lesser extent, nuclear differentiation of the ANP colony from the remainder further suggest a certain degree of genetic isolation and, as the former is mainly due to the presence of a private allele carried by 15.4% of its females, philopatry. The lack of any significant differentiation among the remaining sites indicates either a common, relatively recent origin, and/or high levels of gene flow mediated by dispersal in both sexes. Molecular variance analysis of different grouping designs, which returned higher values of among‐colony variation when ANP was kept isolated and D_O_ and D_R_ were grouped together with MLP and ZJF, further supports this idea. Radiotracking and a few ring‐recovery data indicate movements between all the studied colonies, which could help to explain the lack of differentiation between them (Popa‐Lisseanu et al. 2009). However, the lack of structure at the mitochondrial level should not be attributed to modern‐day dispersal or group formation dynamics alone. The presence of the two most frequent haplotypes in every population and the star‐shaped topology of the median‐joining network both point to a recent population expansion (Fig. 3). Differences between putative original populations could account for the sharp differences in haplotype diversity found between the first and second colonizer groups. Finally, different group formation processes (dispersal for D_O_ vs. budding for D_R_) could also result in similar differences.

### Regional kin structure

We estimated relationships among individuals based on shared nuclear alleles and analyzed the distribution of close kin (*r *>* *0.25) and mother–daughter pairs across the region. The number of females with at least one close relative in the same colony was unexpectedly high for some sites, particularly for the colony in the city park of Seville (MLP). However, it is the number of parental associations found within our complete data set and encompassing the whole area studied that stands out the most with 57.1% of parental associations found to be intracolonial. The complementary 42.9% of these involved females from separate colonies, suggesting still, relatively frequent movements and thus significant gene flow between the colonies. A recent study revealed a negative correlation between wing loading, migration tendency, and the magnitude of genetic differentiation among bat populations (Burns and Broders 2014). Our study sites are at most 150 km apart (MLP to ANP), and previous studies have not only indicated that *N. lasiopterus* can undergo long‐distance migrations, but have also reported important movements in this particular region (Ibáñez et al. 2009; Popa‐Lisseanu et al. 2009). We therefore expected a more even distribution of dyads, reflecting “regional philopatry” (sensu Vonhof et al. 2008). Instead, we found that 33.6% of females (a conservative estimate considering we could not sample all individuals in every colony) stayed in the same colony as their mothers or daughters. While this estimate falls predictably short of that found in colonies of nonmigratory Bechstein's bats, characterized by strict female philopatry (72%; Kerth et al. 2002), it is higher than what was reported in big brown bats (9%; Vonhof et al. 2008), a species with an estimated migratory range of up to 288 km between maternity and winter roosts (Mills et al. 1975).

### Colonization of Doñana National Park

We studied two consecutive colonization attempts of DNP by giant noctules in relation to the three closest known colonies of the species. We found considerable colocalization of female relatives, pointing to a high degree of philopatry and indicating that reported movements do not necessarily result in stable relocations. The lack of differentiation among all the colonies (except for ANP) could be due to the fact that these are too young for any differentiation to become apparent at the mitochondrial level. The formation of new groups or colonies involves the sampling of alleles from one or more parent groups. The degree to which founding individuals are related to one another will influence the genetic variation of the newly formed groups, and consequently the amount of among‐group variation at the population level (Storz 1999). If the formation of the new colony in DNP was the result of random dispersal of females from different nearby colonies, following Slatkin's migrant‐pool model (Slatkin 1977), we would expect the lack of genetic structure we observed. In that case, there may not have been sufficient time for philopatry to counteract this effect. On the other hand, if the new colony was the result of fissioning of closely related females from another colony (propagule‐pool model, Slatkin 1977), the level of genetic relatedness among females of the new group would be higher and the genetic sampling less representative of the whole, increasing among‐group variation. It is important to note that no ringed females (sampled or not) from the initial colonization were ever reported back in the new DNP recolonization group. While the recolonizers of DNP harbor fewer haplotypes than its previous settlers (4 and 8, respectively), an analysis of molecular variance failed to detect an increase in among‐colony genetic variation after the creation of either group. The most parsimonious conclusion is that the Doñana, Seville, and Jerez colonies are relatively recent and related. It is likely that they are the result of an expansion of the natural population of *N. lasiopterus* living in the large area of *Quercus* spp. forest in Cadiz Province, encompassing most of Alcornocales Natural Park (ANP). This hypothesis is in agreement with the star‐like distribution of the haplotype network. Nevertheless, the presence of private haplotypes in all new colonies points to the possibility of genetic additions from other colonies (or regions) apart from an ANP source. In summary, it seems likely that the lack of structure found is mostly due to recent demographic changes, not yet counteracted by the structuring effect of philopatry.

The only previous genetic analysis of the formation of a new group in temperate bats is a study of the tree‐roosting big brown bat (*E. fuscus*) by Metheny et al. (2008). The studied colony fissioned, one group moving to a previously uninhabited area 7 km away from the original colony (Metheny et al. 2008). The authors found higher levels of relatedness in the seceding group than in the prefission one, suggesting that females from matrilines with higher relatedness levels had moved together, a pattern that was interpreted as ensuring the cooperative behaviors needed for group formation (Metheny et al. 2008). We found that average pairwise relatedness within the colonizer groups was nearly twice that of established colonies (Table 6) and four mother–daughter pairs were identified within D_R_, indicating that colony formation in giant noctules does to some extent benefit from the coordinated move of related females. However, the presence of multiple haplotypes among the colonizers, leaving regional genetic structure unaffected, and the generally low pairwise relatedness values indicate a more complex scenario. The question remains open as to which individual‐based considerations – such as proximity to foraging areas, temperature conditions, presence of kin or social partners – underlie the formation of a new group in this species. The presence of unrelated individuals can either be explained by independent simultaneous movements of females, or cooperation and information sharing. Given their flight range (females can cover distances exceeding those between colonies during nightly foraging bouts – Ibáñez et al. 2009; Popa‐Lisseanu et al. 2009), it is reasonable to assume that independent discovery of roosts available at the new site by several females would have been quick. If the site's advantages were clear (i.e., unoccupied bat boxes, overcrowding of the remaining sites, proximity to Doñana's insect‐rich foraging grounds), the arrival by unrelated females might have simply involved their individual choice to move, its speed giving the appearance of one coordinated movement. On the other hand, kinship‐independent information transfer about novel roosts and their relative quality has been reported in Bechstein's bats (Kerth et al. 2002; Kerth and Petit 2005) and could also, if confirmed in giant noctules, explain the simultaneous movement of several females to a newly available area. Our own analysis of parent–offspring dyads involving individuals from both the original and recolonizing groups of Doñana identified an additional six dyads (42% more) in the latter group, the majority of these (9/11) related to females from Seville. Together with the small number of haplotypes in that group and the clustering with MLP in the AMOVA, our results seem to point to a common origin, in support of the latter hypothesis. However, because we are lacking exact information on the initial steps of the colonization, as well as on interactions among the colonizers prior to their movement, the dynamics of this process cannot yet be fully understood. It is possible that for a species of long‐range fliers the decision to switch between colonies within this range is simply not under significant energetic restraints. On the contrary, at least three of the studied colonies (including the one in DNP) may be acting as a large social unit with frequent exchanges between them, despite their distance and the region's habitat heterogeneity (Popa‐Lisseanu et al. 2009).

It is likely that the process of colonization is not a fixed species characteristic, but rather a plastic behavior molded by social and ecological factors. Group fission along matrilineal lines documented for *E. fuscus* by Metheny et al. (2008) is probably not the norm, even within the same species, as suggested by the lack of genetic structure among the populations of big brown bats studied by Vonhof et al. (2008). Even though the existence of a fine‐scale genetic structure has been reported in many mammalian societies (Altmann et al. 1996; Ratnayeke et al. 2002; Nussey et al. 2005; Robinson et al. 2012), suggesting that kinship plays an important role in group choice during group fission, more research is needed to understand the relative roles played by kinship and social bonds (see also Lukas et al. 2005). A predominance of the latter would explain the divergent results obtained across different bat species, in which average relatedness within social groups is remarkably low (Castella et al. 2001; Kerth et al. 2002; present study). We found evidence of philopatry, as well as of cooperation among kin during the formation of new breeding colonies in *N. lasiopterus*. However, the lack of suitable roosting grounds available in this heavily deforested region (Valbuena‐Carabaña et al. 2010) is likely to play a strong role, and could impact the decision to remain with kin (Russo et al. 2010). Moreover, the crash of the D_O_ population in 2007 remains unexplained, but highlights the fragility of any colonization process.

In summary, further investigations into these unique populations will be essential to better understand bat social dynamics as well as help to efficiently design programs for the preservation of this rare and endangered species.

## Conflict of Interest

None declared.

## Data Accessibility

Mitochondrial DNA sequences have been uploaded to GenBank (Accession numbers: HVI: KX709626 ‐ KX709837, HVII: KX621557 ‐ KX621770). Microsatellite genotypes, sample ID and location, R scripts for *F*_ST_, *D*_EST_, and allelic richness calculations were deposited in the Dryad Digital Repository (doi: 10.5061/dryad.rc504).

## Supporting information


**Appendix S1.** Detailed description of DNA extraction, purification, sequencing and genotyping.
**Table S1.** Summary statistics and PCR specifications for microsatellite loci.
**Table S2.** Pairwise D_EST_ values among populations based on microsatellite data.
